# Stigma and self-management: an Interpretative Phenomenological Analysis of the impact of chronic recurrent urinary tract infections after spinal cord injury

**DOI:** 10.1038/s41394-018-0042-2

**Published:** 2018-02-12

**Authors:** Jasmine Heath Hearn, Sen Selvarajah, Paul Kennedy, Julian Taylor

**Affiliations:** 1 The University of Buckingham Medical School, Hunter Street, Buckingham, UK; 2 Stoke Mandeville Spinal Research, The National Spinal Injuries Centre, Buckinghamshire Healthcare NHS Trust, Aylesbury, UK; 3 Department of Clinical Psychology, The National Spinal Injuries Centre, Stoke Mandeville Hospital, Buckinghamshire Healthcare NHS Trust, Aylesbury, UK; 4 Sensorimotor Function Group, Hospital Nacional de Parapléjicos, SESCAM, 45071 Toledo, Spain; 5 Harris Manchester College, University of Oxford, Oxford, UK

## Abstract

**Study design:**

Qualitative, phenomenological design.

**Objectives:**

Neurogenic bladder dysfunction and urinary tract infection (UTI) are common secondary consequences to neurological damage to the spinal cord. This study sought to establish the impact of chronic, recurrent UTIs on people with spinal cord injury (SCI).

**Setting:**

Community sample, United Kingdom.

**Methods:**

Twelve participants with SCI, aged between 28 and 68 years, who had experienced at least three recurrent UTI events within the previous 12 months were recruited. Detailed qualitative information was obtained from semi-structured interviews, which lasted between 30 and 60 min. Interpretative Phenomenological Analysis was performed to explore the lived experience of UTIs.

**Results:**

Interview findings identified a range of factors related to the experience of recurrent UTIs in people with SCI. These were classified into the following themes: (1) Symptom Management Precedence, (2) Stigma-Motivated Risk Management and (3) Exhaustive Exploration of Treatment Options. Participants discussed management of acute exacerbations. Distress arose from perceptions of UTIs as potentially stigmatizing and fear of relying on antibiotics. Arising from this fear, many participants sought alternative prevention and management strategies.

**Conclusions:**

These results suggest that chronic recurrent UTIs act as major barriers to social participation, with adverse effects on quality of life of people with a neurogenic bladder after SCI. People with SCI would benefit from additional assessment of the impact of recurrent UTIs, so that healthcare professionals can address specific concerns, such as the psychosocial impact of urinary incontinence and stigmatizing views. Additional support to enhance self-management and facilitate social participation should be provided.

## Introduction

Each year in the United Kingdom, around 1,200 people are diagnosed with a spinal cord injury (SCI) [1], of which bladder dysfunction and urinary tract infection (UTI) are common secondary consequences [2]. When functioning normally, the bladder stores and expels urine in a coordinated and controlled manner [3]. Following SCI, however, the bladder may not empty efficiently or at all, leading to either over-filling or urinary incontinence (which can also be triggered by spasticity). In turn, this can lead to high bladder pressure voiding, stones in the urinary tract and vesicoureteral reflux [4]. Neurogenic bladder dysfunction can be managed with either timed voiding (using the toilet at regular intervals), intermittent catheterization (4–8 times a day), or with indwelling urethral or suprapubic catheter [4].

People with neurogenic bladder dysfunction associated with inefficient bladder voiding and the use of catheters are at a high risk of suffering from UTIs [5]. On average, people with SCI experience two to three episodes/year which, if left untreated, may develop into a serious secondary complication that can lead to calculi, pyelonephritis, bacteremia and renal failure [6]. Currently, for men and women with uncomplicated UTIs (i.e., no functional or structural abnormalities in the urinary tract), UTI symptoms and signs are identified using guidelines available from the National Institute for Health and Care Excellence [7, 8]. However, given that those individuals with SCI do not normally experience symptoms such as ‘pain’ and ‘a frequent need to urinate,’ the additional diagnostic value of these guidelines are limited for determining the presence of a UTI. Further, the definition of UTI is not consistently defined across studies involving patients with SCI. A recent systematic review indicated that no consistent definition of UTI was followed in 12 reviewed studies [9], suggesting that the screening, diagnosis and prevention of UTI may be inconsistent across clinical research sites and healthcare providers, as well as across those experiencing UTIs directly.

In the present study, an exploration of patient perspectives regarding UTI after SCI was performed using Interpretative Phenomenological Analysis (IPA) [10]. Such an approach allows participants to discuss experiences in their own terms and context, utilizing interpretation to understand ‘what personal and social experiences mean to the people who experience them’ [11]. IPA can illuminate existing quantitative literature by understanding experiences through the accounts of those living with the phenomenon of study and has demonstrated utility for enhancing understanding of other aspects of life after SCI, such as pain [12] and adjustment [13].

To date, no qualitative research study has explored peoples’ lived experiences of UTIs following SCI. Only one published qualitative study was identified in this area and addressed factors that influenced the choice of bladder management for male patients [14], indicating a gap in the literature regarding current understanding of the impact of UTIs, from the patient perspective. This study, therefore, aims to provide useful insights and potential targets for intervention to improve patient-centered care by exploring the subjective understandings and experiences of symptomatic UTIs, if and how these experiences converge and diverge, their management strategies and the psychosocial impact of UTIs on people living with recurrent UTIs after SCI.

## Methods

### Ethical considerations

Local ethical approval was sought from the NHS Health Research Authority (15/LO/2069). Anonymity of data was ensured, following written informed consent. Subjects were reminded of their right to withdraw at any point and opt to have their data destroyed if they subsequently wished not to participate. Written, informed consent was obtained before the semi-structured audio-recorded interview.

### Participants

Inclusion criteria were: age between 18 and 75 years, at least 1 year post SCI, experience of at least three symptomatic UTIs requiring an antibiotic course of treatment within the previous 12 months and ability to provide informed consent. The final sample consisted of 12 participants (6 men and 6 women). Due to the large amount of data obtained and IPA’s detailed, idiographic approach to analysis, this sample size is considered appropriate, in accordance with recommendations of a small sample size for IPA studies by Smith et al. [15]. Ages ranged from 28 to 68 years. Neurological injury level varied from C5 to L2. Participants were between 3 and 47 years post injury. The participants represent a self-selecting sample, such that they had experiential knowledge of the specific phenomenon of study [15]. Pseudonyms are provided to protect anonymity. Participant characteristics are presented in Table 1.

**Table 1:** Demographic and neurological SCI characteristics of recruited cohort

Participant	Gender	Age at time of interview	Marital status	Level of injury	ASIA	Time since injury (years)
Charlotte	F	35	Single	C5	A	14
Louise	F	28	Single	C6	A	10
Sarah	F	62	Married	T7-T8	E	16
Jane	F	60	Married	T11	D	3
Paul	M	62	Married	T12	A	39
James	M	43	Married	L1	C	23
Jack	M	51	Married	T5/6	A	25
Emma	F	60	Married	T4	B	14
Barry	M	68	Widowed	L1-L2	B	24
Dan	M	64	Married	C5-C6	A	47
Guy	M	61	Married	T11	C	43
May	F	31	Single	T5	A	11

### Data collection

A qualitative, single-interview design was employed, recruiting outpatients of a rehabilitation unit. Participants were purposively recruited according to the inclusion criteria, from The National Spinal Injuries Centre, such that they were considered ‘experts’ in the phenomenon of study. People meeting the inclusion criteria according to medical records were identified by treating clinicians who provided them with a letter of invitation. Those interested were directed to the researchers, provided with detailed information and given time to consider their participation. Of the 27 people invited to take part, 12 consented. Interviews were arranged at times suitable to the participant and coincided with scheduled appointments at the treating centre, thus allowing for interviews to be conducted face-to-face. Written, informed consent was obtained before the semi-structured audio-recorded interview, which were conducted in private rooms in the hospital, and lasted between 30 and 60 min. The interview schedule, composed of open-ended questions, was developed with contributions from all authors, who have experience of working with people with SCI, with the aim to use this only to elicit further information from participants where necessary. Interviews consisted of open questions referring to the experience of UTIs, related symptoms, and emotional responses. Each interview began with ‘tell me about your experience of UTIs snce you sustained your SCI’. Participants were given freedom to lead the interview, and were encouraged to discuss what was important to them, such that the data obtained were based on the participants’ personal contexts, as opposed to the imposition of topics. The full interview schedule is presented in Table 2.

**Table 2:** Interview schedule

1) Tell me about your experience of UTIs since you sustained your SCI
• What is your first sign? What is the frequency and duration of this?
• Are there any other signs and symptoms? (e.g., mood, discomfort, pain, temperature?)
• What is the physical impact of your UTI, if any?
• Which of your symptoms are most important to you in terms of determining whether you have or are about to have a UTI?
• When you have a UTI, how long does it generally take to reach a diagnosis?
• What impact does it have on you and your rehab?
2) Your emotions can have a significant role in your experience of UTIs. Have you recently experienced any psychological difficulties or concerns?
• If so do you think this has influenced your experience of your UTIs?
• Impact on mood, rehab, symptoms?
• What do you do to manage your UTI, frequency and duration?
3) What is your chosen method of catheterization and who taught you your chosen method?
• Do you fully follow the required procedure of catheterization (e.g., washing hands)?
• To what extent does this impact your life?
• How do you feel about this method of catheterization?
4) Who do you present your symptoms to? What normally happens?
• How does your healthcare professional respond to and manage your UTIs?
5) What do you do to prevent UTIs?
• How did you learn about these techniques?
• How helpful are these strategies?
6) What would you suggest to someone who has just been diagnosed with a UTI?
7) What would you suggest to healthcare professionals to improve their care and management of UTIs?

### Data analysis

Interviews were transcribed verbatim. Any identifying information was changed at the point of transcription to protect the identities of participants and any other identifiable people and places. As its aim aligns with that of the present study (to explore subjective experience), IPA [15] was adopted as the most appropriate methodology. Numerous readings of the transcripts were conducted, followed by the development of emergent themes by coding descriptive, linguistic and conceptual notes on a line-by-line basis, as recommended by Smith et al. [15]. In-depth interrogation and interpretation of the participants’ experiences and the interrelationships between cases led to the development of super-ordinate themes with quotes to illustrate the content of themes.

Each case was analysed separately before moving to the next, to ensure that themes remained grounded in the participant’s account. Upon completion of individual analyses, a cross-case analysis was performed, with convergences and divergences identified. Identified themes were cross-checked with corresponding quotes, to ensure that theme labels were grounded in, and reflective of, the data. The interpretative nature of IPA suggests that individual researchers are likely to interpret data differently, according to personal contextual backgrounds. Determined efforts were therefore made to ‘bracket off’ prejudgements and information learned from previous interviews. The use of a reflective diary and dedication to individual participant accounts aided the researchers to remain true to participant experiences. Two independent auditors were enroled, to ensure rigor in the quality of the analysis. Following analysis of each transcript, auditors checked the super-ordinate themes and corresponding quotations from each interview to ensure themes were grounded in the data in the form of a matching exercise. Thoughts and alternative interpretations of theme titles and quotes were discussed to illuminate areas of the experience that may have been more easily identifiable to them. Following completion of the cross-case analysis, auditors were presented with quotations from each theme, without the superordinate theme title, and asked to consider their interpretations of the theme. These interpretations were discussed and superordinate themes agreed. The interpretations presented here are considered credible and meaningful, although it is acknowledged that these are not the only possible interpretations of the data [15]. Sufficient data are included to validate themes and interpretations.

## Findings

Three super-ordinate themes arose from the data: (1) Symptom Management Precedence, (2) Stigma-Motivated Risk Management and (3) Exhaustive Exploration of Treatment Options. These themes reflect the complex nature of living with and managing recurrent UTIs. Each theme and each corresponding sub-theme is presented in Table 3 and discussed in further detail below.

**Table 3:** Superordinate themes and corresponding subordinate themes

*Symptom Management Precedence*	*Stigma-Motivated Risk Management*	*Exhaustive Exploration of Treatment Options*
Erratic and unpredictable onset	Emotional overlay	Trial and error of alternative intervention
Proportionate responding to acute exacerbation	Risk management of social participation	Antimicrobial resistance anxiety

### Symptom management precedence

#### Erratic and unpredictable onset

During their interviews, all participants took time to describe the distressing nature of their key symptoms, which were often described as erratic and presenting without pattern or predictability:

Within 24 h I will change quite considerably, I become ill quickly. For me, that means feeling dizzy, feeling sick, having really acute back pain, having frequency, the urethra burns, and it’s very painful, and then the urine starts getting really thick and really smelly. (Sarah)

I find it depressing really, and I’m not actually a depressive person. I struggle to live like this, constantly on the watch for another one, because you can’t predict it. (May)

These descriptions served to aid understanding of the experience, but also to point to the extreme and sudden onset of the symptoms and development of UTIs. This indicates the distressing nature of UTIs and the following requirement to turn focus towards management and away from daily living in a short space of time.

#### Proportionate responding to acute exacerbation

Many others went on to describe events when their symptoms were acutely exacerbated and their responses to such exacerbations. James described one such occasion, which led him to seek emergency help:

I recently had a severe UTI. It was the first time I’ve had it where I actually had a really painful abdominal cramp that went with it, almost felt like my bladder was being torn outside my body. A really horrible tearing sensation, and I actually went to A&E … that was probably the worst sensation I’ve ever had.

Unsurprisingly, the presence of a variety of symptoms, and specifically the pain associated with UTIs, caused psychological distress and fear. James comments on actually going to A&E, demonstrating recognition of the extreme (but in his eyes, proportionate) response to the pain induced by his UTI. Indeed, others described feeling as though they were limited with respect to their options for help-seeking if their general practitioners or consultants were unavailable during acute exacerbations (for example, on weekends).

### Stigma-motivated risk management

#### Emotional overlay

The possibility of incontinence that could accompany UTI also impacted the wellbeing of participants. For some participants, UTIs were accompanied by a strong emotional overlay:

It makes you very low. It’s curious how depressed you can get so very quickly when that UTI hits. Then when you’re very depressed and low, it’s very difficult to feel that you can manage. (Guy)

UTIs had a transformative effect on Guy’s mood, resulting in feeling low, which then culminated in a perception of difficulty in managing an infection. This perception resonated with others and could contribute to deterioration in physical and mental health, should it translate into avoidance behaviours.

For Emma, recurrent UTIs left her with a constant fear of incontinence, especially during social circumstances. Her worries manifested in a felt stigma; a view that being ‘found out’ would lead to punishing responses from others. This had a clear impact on her sense of identity:

I don’t want to be that wee-smelling cripple in the chair, don’t want to be that person. You know, that perception of being disheveled and dirty and smelly and not very pleasant. Nobody wants to have a smelly person in their surroundings. (Emma)

Emma considered her symptoms to reflect ‘abnormal’ behaviour (i.e., being a ‘smelly person’), which led to concerns surrounding her identity. The risk of UTIs becoming ‘visible’ to others around her presented Emma with a fear of being be ‘outed’ and treated differently because of her symptoms. The possible impact upon her identity could lead to reluctance to engage in social situations so as to avoid judgement from others.

#### Risk management of social participation

As demonstrated by Emma in the previous theme, the unpredictability of UTIs fueled fears of social participation. Louise echoed this, describing the necessitated additional planning and consistent bladder management routines:

I’m extra sure that I’ve got spare catheter changes, extra incontinence sheets, spare changes of clothes. If I can, I won’t go too far from home… I’m always prepared anyway. I’ve always got a spare change of clothes, always got spare medication, always got everything. (Louise)

Louise described micromanaging her life around her bladder and UTIs, which prevented her from fully engaging in social activity without fear. Since experiencing recurrent UTIs, determined efforts were made to prepare for any eventuality to reduce the risk of social judgement, while maximizing participation in daily life as far as possible. However, the heightened risk of urinary incontinence acted as a barrier to spontaneity, freedom and independence, which Sarah also emphasized:

My UTIs control my life, my bladder controls my life. Every time I go out of the house my bag has to be packed with all the pads, the catheters, everything … It’s something that is out of my control, and when something is out of your control, it can make you feel really quite low, because you can’t self-help. You start to feel helpless. (Sarah)

For Sarah, UTIs were life-dominating; her life revolved around attempts to maximize her sense of control when experiencing a current UTI and her preparation for them at any moment. The lack of ability to be spontaneous without making backup plans resulted in feelings of helplessness and loss of control, thus impacting upon her psychological wellbeing.

### Exhaustive exploration of treatment options

#### Trial and error of alternative intervention

Participants described considerable interest in alternative methods of managing or preventing UTIs, despite their varied efficacy: ‘I’ve tried cranberry, waterfall d-mannose, all of that stuff and it just doesn’t work for me.’ (James). Potentially arising from the inefficacy of the commonly tried techniques, unique methods had been explored: ‘I eat more live yogurt for the probiotics, but I don’t know how it might work. I haven’t noticed anything different since I started five months ago.’ (James). Similarly, others continued using complementary methods, though no obvious benefit was noted:

I have a probiotic drink every day … I take D-mannose, I don’t know if that works, and I also take cranberry tablets daily. I don’t know whether any of those help and I’m in that thing where I don’t want to stop taking them in case they do help. I was told that each one of those things is supposed to help but whether or not they really do … I’m nervous about stopping taking them. (Emma)

For Emma, adhering to complementary methods for UTI prevention was motivated by the hope for some yielded benefit, no matter how small and regardless of whether she noticed any benefit. Her quote also exhibits anxiety around reducing adherence to complementary methods, demonstrating her desperation to avoid UTIs at any cost. This theme highlights the distress caused by UTIs and the fearfully motivated continual search for alternative techniques.

#### Antimicrobial resistance anxiety

For many participants, the risk of physiological resistance to antibiotics was a prominent issue, enhancing motivation to seek out alternative methods:

They always try and give me antibiotics first. I’m like ‘no, no, no’ … I think they kill you as much as they cure you. Often, they make me feel worse before I feel better. If I can fight a UTI naturally, I will try … they make me feel quite vulnerable … I’d like a magic pill, that’s going to stop them from happening. Unlikely, [but] I’d like the fear of resistance to antibiotics not to be there. (Louise)

Louise discussed being given antibiotics as the only option to manage her UTIs. These, however, often made her feel worse, prolonging the time taken to recover, which subsequently led to the development of fears of increased risk of antimicrobial resistance. Consequently, Louise demonstrates an aversion to antibiotics, manifesting itself in the desire for a ‘magic pill’ to reduce the risk posed by recurrent antibiotic use.

For others, physiological resistance was already present, increasing anxieties around mortality:

The anxiety for me is, is this antibiotic going to work? The big thing for me is the anxiety over the resistance getting worse. Then I feel ‘is this it? Am I going to go this week?’ … it makes me confront my mortality a lot. (Jane)

I’m not very pro-drugs at all. I’d rather not take antibiotics unless I need it. I don’t want to put words in my doctor’s mouth but he seemed pretty set in what he said, that the antibiotics aren’t working in the long-term … I’m obviously concerned. (Paul)

For those already displaying signs of antimicrobial resistance, the reality of their mortality was prominent in their minds, with concerns reinforced by medical confirmation of resistance. The combination of antimicrobial resistance and increased risk of UTIs after SCI leaves these patients at risk of further health complications, particularly if antibiotics remain the first response to such infections. This underscores a key concern for those with SCI and recurrent UTIs.

## Discussion

This is the first published qualitative study to explore the experience of recurrent UTIs after SCI. This study found that there are a number of challenges posed by UTIs and of living with a neurogenic bladder more generally, confirming the profound impact upon the lives of individuals with SCI. These challenges emerged in three super-ordinate themes: (1) Symptom Management Precedence, (2) Stigma-Motivated Risk Management and (3) Exhaustive Exploration of Treatment Options, which serve to highlight the extremities of symptoms experienced, the impact of UTIs on social participation and identity, and the desperation with which people with SCI seek alternative options for UTI management. These themes are not uncommon following SCI; Burns et al. [16] explored the experience of neurogenic bowel dysfunction after SCI and yielded similar results. The present study confirms that UTIs are subjectively distressing and require complex management that incorporates consideration of the psychosocial impact.

Participants described feelings of anxiety and depression when UTIs were present and expressed concerns regarding discrimination if symptoms became obvious to others, specifically in the context of daily living and social participation. This resulted in taking specific steps, to ensure they were as prepared as possible for any eventuality, such as staying close to home. Despite this preparation, however, the increased risk of symptoms such as urinary incontinence limited their ability to be spontaneous in everyday life. Similarly, negative stereotypes surrounding being a wheelchair user led some participants to take measures to isolate themselves from others. Such behaviours arose from a ‘felt’ stigma; a concern that being ‘outed’ by UTI symptoms would lead to differential treatment, discrimination, or other unaccommodating behaviour, should UTI symptoms become apparent. Such feelings will likely have implications for social participation and work lives, as well as psychological wellbeing. This result supports previous work indicating that UTIs have adverse effects on the health [17] and quality of life of people with SCI [18]. The present study suggests that improved management of symptoms and the reduction of stigma around UTIs, and being a wheelchair user could improve social participation and wellbeing. Similarly, assessing the individual impact of UTIs on psychological wellbeing and social participation, as well as the experience of living with a neurogenic bladder more generally could provide healthcare providers with valuable insight into the impact of UTIs, and help guide educational intervention to aid self-management.

As the first line of treatment, concerns around antimicrobial resistance arising from repeated use of antibiotics were identified. Currently, antibiotics are crucial to the management of UTIs, but inappropriate use and/or overuse can lead to antimicrobial resistance and side effects such as allergies and bowel disturbance. In line with concerns expressed by participants in the present study, innovative approaches to the prevention of UTIs have begun to be explored, such as using probiotics [19]. Probiotic intervention protocols using an avirulent Escherichia coli strain to prevent symptomatic infection with virulent strains, found a small benefit to those with SCI in terms of reduced number of UTIs [20]. E. coli has been identified as the most common cause of UTIs after SCI, with around 60% of UTIs in community dwelling people, and 44% of those in hospital caused by this organism [21]. Another study explored the effect of aerobic physical training, in which the prevalence of chronic asymptomatic bacteriuria decreased [22]. Managing patient concerns regarding antibiotic resistance and the risk of mortality if they are unable to treat UTIs, as well as optimizing self-care, should be addressed.

Many participants described engaging with a trial-and-error process to seek out alternative options for UTI prevention and management of both infection and neurogenic bladder, potentially arising from fears of antibiotic resistance and the impact of UTIs on social participation. Many participants explored bladder management options independent of standard medical care, opting for herbal and complementary remedies, such as probiotic yogurt consumption and cranberry tablets, despite limited effectiveness. Although trials have shown benefit of the topical application of probiotic microorganisms [23], there was no evidence found to support probiotic yogurt consumption for the reduction of UTIs. Similarly, mixed evidence exists for the use of cranberry tablets; one controlled trial showed no reduction in UTIs vs. placebo [24], whereas another found significant reductions [25]. Participants in this study actively sought out alternative options, often independently of their healthcare providers, which may offer an increased sense of control. This suggests that those involved in the care of people with SCI and recurrent UTIs should acknowledge attempts to self-care, whilst providing further guidance and support regarding safe and efficacious alternative interventions for bladder management.

### Limitations

Caution should be exercised in generalizing the results due to the small sample. Further, participants openly discussed their experiences, and as such, the results are representative of those willing to discuss their UTIs and may not be reflective of the experiences of those unwilling to discuss their experiences. The results of the present study may reflect the experiences of those who find their UTIs particularly distressing. Future work may consider conducting interviews with patients with fewer UTIs, which may establish alternative, and potentially more effective, methods of management and coping not discussed by participants in the present study.

## Conclusions

The results of the present study suggest that UTIs after SCI are a source of physical, emotional and social distress and disruption. However, the depth of understanding obtained from the present study supports previous literature indicating that UTIs have a broader impact than that related to physical issues alone. This information can be used to improve the services provided by those involved in care of people with SCI as well as the treatment of UTIs. The findings provide subjective support for continually improving the availability, reliability and effectiveness of psychological and social support for those with recurrent UTIs, which will likely have an important role in reducing the stigma and improving the wellbeing of those living with SCI and recurrent UTIs.
